# 

**Published:** 1883-07

**Authors:** 


ORIGINAL COMMUNICATIONS:

On the Density of Enamel. J. Edw. Line, D. D. S. .......... 349
Central Dental Association of Northern New Jersey .......... 354
Diseases of the Dental Pulp. Bodecker .......... 369

ORIGINAL TRANSLATIONS AND ABSTRACTS:

Dental Prosthesis .......... 371
A Chronic Complaint .......... 374

EDITORIAL:

The Blessed Doctrine of Rest .......... 375
The American Medical Association .......... 378
Toothache .......... 379
A Change .......... 380

NEW APPLIANCES AND MATERIALS:

The Sandpaper Disc Cutter .......... 380
Robinson's Fibrous and Textile Metallic Filling .......... 381

BIBLIOGRAPHICAL:

Webb's "Notes on Operative Dentistry" .......... 381
An Anomaly of the Human Heart .......... 382

CURRENT NEWS AND OPINION:

Good Advice to Travelers .......... 383
New Jersey State Dental Society .......... 384
The American Dental Association .......... 385
American Society of Microscopists .......... 385
American Dental Convention .......... 385
Notice to State Boards of Examiners .......... 386
Maine Dental Society .......... 386

SELECTIONS:

Dropsy of the Antrum .......... 386
Senses in New-Born Infants .......... 388
Syphilis in the Ninth Century .......... 389

POPULAR SCIENCE DEPARTMENT:

Archeological Frauds .......... 390
Baldness .......... 391
The Latest Theory of Sea-Sickness .......... 392
Impregnation in the Turkey .......... 393
Carbolic Acid as a Local Anæsthetic .......... 393
Properties of Saliva .......... 394
Potatoes as Pen Wipers .......... 394
Incubation of Infants .......... 394
Localization of Functions in the Cerebral Cortex .......... 395
Melting Gold .......... 395

